# Adaptive Controller Based on Spatial Disturbance Observer in a Microgravity Environment

**DOI:** 10.3390/s19214759

**Published:** 2019-11-01

**Authors:** Chunguang Fan, Zongwu Xie, Yiwei Liu, Chongyang Li, Hong Liu

**Affiliations:** State Key Laboratory of Robotics and System, Harbin Institute of Technology, West Dazhi Street, Harbin 150001, China; jerry_chunguang@163.com (C.F.); lyw@hit.edu.cn (Y.L.); lichongyang@stu.hit.edu.cn (C.L.); hong.liu@hit.edu.cn (H.L.)

**Keywords:** space manipulator, central controller, gravity compensation, spatial disturbance observer, friction compensator

## Abstract

In this paper, a new controller for an operating manipulator work in the space microgravity environment is proposed. First, on the basis of the load variation caused by microgravity, a sliding mode control method is used to model the gravity term, and the logistic function is introduced as the approaching function. An improved sliding mode reaching law is proposed to control the manipulator effectively, and Lyapunov theory is used to deduce its closed-loop stability. A friction compensation scheme, which regards friction as disturbance, is introduced to the microgravity environment, and a space disturbance observer (SDO) is designed from the viewpoint of disturbance suppression to identify the friction characteristics of the control system accurately. To model the lagging friction phenomenon caused by velocity inversion during operation tasks, an adaptive compensation scheme based on the LuGre model is proposed. Finally, the design of a manipulator system, which consists of a robot arm, dexterous hand, teleoperation system, central controller, and visual system, is presented. On-orbit maintenance and capture experiments are carried out successively. The effectiveness and reliability of the controller are verified, and the on-orbit operation tasks are completed successfully.

## 1. Introduction

Space technology is the realization of human’s infinite yearning to explore the universe. With the continuous exploration of the space environment, the necessity of space robots for spacecraft maintenance is growing with the increase in captured space objects and various space experiments. However, there are two contradictions in the operation of space robots: they come from an uncertain space environment to carry out high-precision tasks, and there are inconsistencies in task requirements that result from the difference between space microgravity and ground gravity environments. The change in mechanical motion behavior and control performance caused by the microgravity environment has become an unavoidable problem in the design of space technology. Because of the space microgravity environment, the ideal model is entirely different from the actual model, and this divergence seriously affects the final control accuracy of the space robot manipulator. The dynamic equation of the manipulator consists of three parts: inertia term, Coriolis and centripetal force term, gravity term, Therefore, an effective control strategy is needed to preprocess the gravity term while compensating for the friction of the space manipulator to account for the differences between space and ground conditions.

Nowadays, a lot of space robots developed to help astronauts completing various operating tasks, such as the NASA/DARPA’s space astronauts plan, which design and develop the famous robot named Robonaut, in order to realize the robot astronaut extravehicular activity. It uses the master-slave teleoperation method to complete all kinds of the interactive task [1,2], the Cog project, an upper-body robot with more complex intelligent sensors is designed to tosimulate and explore human behavior [3], at the same time, an integrated multi-sensor humanoid robot with long-lasting batteries Dav [4] was developed by the MSU. MIT’s Domo robot can perform operational tasks in an unknown environment [5], In addition, Justin [6], a robot developed by DLR, demonstrates a richer range of two-handed tasks in daily life scenario, by adopting a dynamic compensation controller in the real system for the first time [7]. In terms of extra-vehicular applications, the space teleoperation manipulator can be used to complete the deployment and recovery of satellites, as well as check the working conditions of the spacecraft’s temperature protection system and a series of complex tasks [8], the SPDM [9] manipulator can complete a variety of extra-vehicular tasks, and adopts a new system design method to reduce the cost by 30%, which provides a new idea for the research and development of the space manipulator with low-cost and high efficiency in the future. In keeping with this cost-cutting approach, the DARPA created the Orbital Express program [10], which aims to develop a variety of orbital infrastructure capable of autonomous, low-cost orbital refueling, delivery of small payloads and other complex tasks. In the ROTEX project [11], key technologies such as multi-sensor acquisition technology, local perception compensation technology and prediction simulation are adopted, which proves that the adoption of this human-machine configuration system is an important solution for human-machine cooperative operation in the future.

Friction is one of the main factors limiting the desired performance of a manipulator’s maintenance task [12] because it not only affects the static performance and dynamic performance of the manipulator but also leads to limit cycles, steady-state errors, and even instability. Especially in low-speed missions, the performance of the control system is seriously reduced by the lack of friction compensation or inaccurate friction compensation, leading to mission failures. To prevent these friction-induced limitations, many methods have been used to minimize the adverse effects of friction. The most basic method is to compensate for friction using models. The friction model can be divided into two categories:

- Static friction models, such as the Column–Viscous model and the Stribeck model.
- Dynamic friction models, such as the Dalh model [13], LuGre model [14], etc.

Model-based methods depend on the accuracy of the model, the identification of model parameters, and the realization of a friction compensator. A proper friction model is the foundation of achieving good compensation results.

Other friction compensation methods do not use models, so the accuracy of the friction model is not a concern. The sliding mode controller is the most simple of such model-free methods. For example, friction is usually considered a bounded disturbance signal, and a control strategy enables the tracking error converge to zero. The advantage of this kind of method is that the degree of accuracy required for a friction model is not a consideration. The main disadvantage of this kind of control method is the difficulty in overcoming the control signal vibration, which reduces the life of mechanical parts and has a severe impact on system reliability. Another method uses the nonlinear friction term as the disturbance; for example, the traditional PI servo controller of integral action can work around the target location of the limit cycle. If we adopt PD control, the friction leads to a finite steady-state error that can be indirectly decreased by improving the gain of the tracking error to reduce the friction force and the inertial force of impact; however, this increases the instability of the closed-loop system. The traditional PID controller can meet the basic requirements in the field of industrial control, but it is unable to meet the requirements for control stability in space missions. At the same time, the control accuracy of the dynamic response is desirable. On the other hand, the mechanical manipulator in all space missions is subject to nonlinear friction. In a high-performance control system, nonlinear friction is inevitable because the performance of the mechanical manipulator’s static characteristic is based on the tracking error and oscillation of the limit cycle; the performance of the mechanical manipulator’s dynamic features is based on lower creep and a waveform distortion rate that reaches zero.

### 1.1. Related Work

#### 1.1.1. Friction Compensation

Wen-Hua Chen [15] proposed a new nonlinear disturbance observer (NDO) by selecting the design parameters that would ensure the disturbance observer’s global exponential stability, which would overcome the shortcomings of the existing disturbance observer. J. Swevers [16] proposed a new dynamic friction model structure that handles the Stribeck effect and stick-slip behavior, and De Wit C C and Lischinsky P [17] studied an adaptive friction compensation model on the basis of the dc servo motor application. First, they proposed a two-step offline estimation method for the nominal static and dynamic parameters of the model. Canudas d W C [18] analyzed the modeling and compensation problem of the friction velocity approaching zero. Simple models (such as the Coulomb friction model) have been used as the basis of friction compensation and combined with the adaptive torque calculation method. Yoo B.K. and Ham W.C. [19] proposed two adaptive control schemes for a manipulator with parametric uncertainties. A fuzzy logic system (FLS), which can approximate any nonlinear function in a compact input space, was used to compensate for these uncertainties. In their proposed control scheme, it was not necessary to derive a linear equation of robot dynamics. In reference [20], a tracking controller was proposed by C. Makkar, G. Hu, and W. G. Sawyer for a class of Euler–Lagrangian systems with continuously differentiable friction models with uncertain nonlinear parameter terms. In reference [21], an adaptive controller based on a sliding mode observer was proposed by Xie W F and Zhao Z Y for friction servo actuators. Lu L, Yao B, and Wang Q [22] introduced a new friction model that is equivalent to the LuGre model at low speed and the static friction model at high speed, with a continuous transition between the two. Jin M and Kang S H [23] proposed a simple, robust, and compliant control method for a manipulator with nonlinear friction. A new neural network structure was introduced by Selmic R R [24]. Their proposed neural network could approximate any piecewise continuous function with a discontinuity at a finite number of known points by using new neurons with a “jump approximation” activation function. This augmented neural network was used for friction compensation. Armstrong B, Neevel D, and Kusik T [25] adopted NPID control, which was realized by changing the controller gain as a function of the system state. Yao J, Deng [26] improved the traditional piecewise continuous LuGre model, deduced a new continuous, differentiable nonlinear friction model, and then proposed an adaptive backstepping controller to manage the parameter uncertainty of hydraulic systems. A robust friction compensation scheme was proposed in [27]. A recursive fuzzy neural network (RFNN) and a reconstruction error compensator were used to develop a robust frictional state observer. Han M K, Park S H, and Han S I [28] studied the tracking control problem of a mechanical servo system with nonlinear dynamic friction and proposed a composite control scheme consisting of a friction state observer, an RFNN approximator, and an error compensator with sliding mode control. A new friction model was also proposed by Makkar, Dixon [29]. W. Lee [30] propose a friction observer based on a Kalman filter with load estimation for friction compensation control considering the applied load change. S. Wu [31] develop a friction observer that can effectively estimate the friction torque.

#### 1.1.2. Sliding Mode Control

Draenovi, B [32] proposes an effective method to reduce the sensitivity of parameter changes and disturbances, the proposed method does not need to measure disturbances or identify objects. Considering the change of the model due to the change of gravity environment, Li, Qin [33] adopted a robust adaptive control strategy for trajectory control. A continuous finite-time control framework was proposed by Yu, Shuanghe [34], and a terminal sliding mode control method was used to obtain good track tracking performance and fast response time. Guo, Yuzheng [35] proposed an adaptive fuzzy sliding mode controller, which has well-releasing vibration characteristics compared with classical sliding mode control. Hashimoto [36] proposed a sliding mode control method based on the variable structure system with simple nonlinear compensator, which is very suitable for online computer control. Islam [37] describes that when faced with a large range of uncertain parameters, a multi-model sliding mode control observer is proposed to reduce the observer-controller gain and effectively suppress the tremor. Jafarov [38] proposed a new variable structure controller, which did not need to use equivalent control terms and introduced saturation function to deal with the chattering phenomenon. Nazari [39] uses artificial intelligence theory and parallel fuzzy logic theory to compensate the dynamic uncertainty of the system, and solves the problem of nonlinear equivalent equation and chattering phenomenon in the uncertain system. In order to realize high robustness and high precision position tracking, the sliding-mode control scheme inherited by the fuzzy neural network was proposed in [40], the adaptive tuning algorithm of network parameters was deduced, and the control performance of convergence and stability was achieved. Chen [41] proposes a sliding mode control method of robot neural network based on sliding mode variable structure control and neural network convergence law. The chattering phenomenon in sliding mode control is eliminated through the adaptive adjustment of parameters of two feedforward neural networks. Capisani [42] adopts a robust force and position hybrid control scheme, which uses the first and second-order sliding mode controllers to generate robot input rules, and realizes the interactive control strategy in the presence of unknown obstacles. Literature [43] researches the state estimation and sliding mode control problems for phase-type semiMarkovian jump systems, establishes sufficient conditions for the solvability of the desired observer. Literature [44] designs an integral sliding mode surface and observer based adaptive sliding mode controller.

#### 1.1.3. Disturbance Observer

Literature [45] proposes a finite time control method for an uncertain nonlinear system, design an adaptive sliding mode disturbance observer to estimate the disturbance in finite time, and based on the output of proposed distubance observer, and presents a terminal sliding mode control scheme for the uncertain nonlinear system. Literature [46] proposes a disturbance-observer- (DO) based PBC (DO-PBC) for static synchronous compensator (STATCOM), which provides faster responses in handling various kinds of disturbances. Literature [47] proposes a novel coordinated control strategy, and the frequency and voltage can be regulated by taking advantage of adaptive sliding mode (SM) method and disturbance observer (DOB). Literature [48] presents a model-based friction compensation method combined with an observer-based adaptive sliding mode controller for the speed loop of electromechanical actuator system, and discuss the stability of system with Lyapunov stability theory and Barbalat’s lemma. Literature [49] studies a novel speed control method which adopts the single-loop control structure based on sliding mode control and disturbance observer, and designs a multiple-surface sliding mode controller. Literature [50] proposes a prediction-based super twisting sliding mode control (ST-SMC) using a state and disturbance observer.

### 1.2. Significance of This Paper

In previous studies of mechanical manipulators, a static or dynamic model was used to describe all friction behavior. In such models, the friction cannot adaptively change in response to variation in the interaction mode between the controlled target and the contact environment. In the space microgravity environment, friction is often accompanied by pre-sliding and sliding, which are two frequently switching states. So, first, this paper presents a new design of a space disturbance observer (SDO) that was developed using a disturbance observer. This paper then proposes a new friction model to compensate for the friction. The proposed model allows the target to switch adaptively between the sliding and pre-sliding states. Next, an adaptive friction compensation controller based on the spatial disturbance observer is proposed. This controller can realize precise adaptive friction compensation for the operation of a manipulator in the microgravity environment.

Following the guideline in [16], a controller architecture for the space microgravity environment was designed. The controller is composed of the SDO, friction compensator, and gravity preprocessor. According to the load changes caused by the space microgravity environment, sliding mode control is used to preprocess the gravity items, and a logic function is introduced as an approximation function. An improved sliding mode approximation method is proposed to control the manipulator effectively, and Lyapunov theory is applied to the closed-loop stability analysis. A friction compensation scheme based on friction in the microgravity environment is proposed. The SDO was designed to accurately identify the friction characteristics of the control system from the angle of disturbance suppression. An adaptive compensation scheme based on the LuGre model is proposed for the lagging friction caused by the speed reversal that occurs during the implementation of the task. A new humanoid manipulator system, TG2, was ultimately designed; it consists of a manipulator, a dexterous hand, a teleoperation system, a central controller, and a visual system. On-orbit maintenance and acquisition experiments were carried out successively using this system. The validity and reliability of the controller were verified, and the on-orbit operation task was completed successfully.

The main structure of this paper is as follows: The second section introduces the system dynamic model, methods of dealing with the gravity term, and the friction compensation strategy. In this section, the stability of the sliding mode control algorithm used in gravity compensation is demonstrated. The third section introduces the composition and construction of the experimental platform so that readers understand the context and significance of the follow-up experiment. Then, the design of two relatively independent experiments is presented. The experiments were designed using the control scheme proposed in this paper. The fourth section discusses the experimental results. Finally, the conclusions and prospects of this research are given.

## 2. Materials and Methods

In this study, the manipulator component of the TG2 manipulator system was used as an algorithm verification platform. The main task of the platform is to complete the maintenance operation and perform designated tasks. In Section 4, the design and composition of the platform are briefly introduced. This section introduces the dynamic model of the manipulator and the gravity preprocessing method in the space microgravity environment, and then friction compensation is presented.

### 2.1. Dynamic Model

The main problem faced in the debugging process of space robots is the difference between the physical model and the actual environment. Controllers designed for creating models of the debugging process cannot be directly used in the space environment, so the debugging work of space robots on the ground is troublesome. Especially for tracking, positioning, capturing, and other control tasks, the space microgravity environment changes not only the gravity load but also other behaviors of the robot as a consequence of changes in gravity load.

#### 2.1.1. Ground Debugging Model

The basic dynamic equation of the mechanical arm model can be expressed as follows

(1) $$D\left(q\right)\ddot{q}+C\left(q,\dot{q}\right)+G\left(q\right)=\tau$$

where $D\left(q\right)\in R^{n\times n}$ denotes the inertia matrix of the system, $C\left(q,\dot{q}\right)\in R^{n\times n}$ denotes the centrifugal force and the Coriolis force matrix, $G\left(q\right)\in R^{n}$ denotes the gravity load vector matrix, $q={\left[q_{1},q_{2},\ldots q_{n}\right]}^{T}\in R^{n}$ represents the joint angle of the manipulator, and $\tau ={\left[\tau_{1},\tau_{2},\ldots \tau_{n}\right]}^{T}\in R^{n}$ represents the torque of the manipulator joint.

As the base of the manipulator described in this paper is fixed on the installation wall of the space experimental cabin, and various random disturbances will occur during the orbit of the experimental cabin, the dynamic model of the manipulator is described in the following equation during ground debugging

(2) $$D_{0}\left(q\right)\ddot{q}+C_{0}\left(q,\dot{q}\right)\dot{q}+G_{0}\left(q\right)+N\left(q,\dot{q},t\right)=\tau$$

where $D_{0}\left(q\right),C_{0}\left(q,\dot{q}\right),G_{0}\left(q\right)$ are the nominal values of the model, and $N(q,\dot{q},t)$ is the total disturbance of the system.

#### 2.1.2. Space Experiment Model

When the manipulator is on-orbit state, due to the action of microgravity, the dynamics model of the manipulator can be expressed as follows:

(3) $$D_{0}\left(q\right)\ddot{q}+C_{0}\left(q,\dot{q}\right)+N\left(q,\dot{q},t\right)=\tau$$

The variables in Equation (3) have the same meaning as those in the ground debugging model.

The space microgravity environment has a great influence on the mechanical system. Due to the change of the environment, the gravity compensation failure, friction compensation failure, debugging becomes difficult, and the nonlinear disturbance of the dynamic model is increased. Therefore, it is necessary to design a control strategy that can compensate for the adaptive dynamic characteristics in different gravity environments without changing the controller architecture and controller parameters. Therefore, although the gravity term cannot be removed directly, it can be preprocessed using sliding mode control.

### 2.2. Design of Controller

#### 2.2.1. Pretreatment of Gravity Term

Firstly, the position error and joint differential of the manipulator joint are expressed respectively

(4) $$e\left(t\right)=q_{d}\left(t\right)-q\left(t\right)$$

(5) $$\dot{e}\left(t\right)={\dot{q}}_{d}\left(t\right)-\dot{q}\left(t\right)$$

(6) $$\ddot{e}\left(t\right)={\ddot{q}}_{d}\left(t\right)-\ddot{q}\left(t\right)$$

where $q_{d}\left(t\right)$ and q(t) are the expected and actual joint position vector, respectively. Considering the general method of dynamic system modeling, the sliding surface shown below is selected:

(7) $$s\left(t\right)=K_{P}e\left(t\right)+K_{I}\int_{0}^{t}e\left(\tau \right)d\tau +K_{D}\dot{e}\left(t\right)$$

where $K_{P}$ is the proportion gain matrix, $K_{I}$ is the integral gain matrix, and $K_{D}$ is the differential gain matrix.

The derivative of Equation (7) is

(8) $$\dot{s}\left(t\right)=K_{P}\dot{e}\left(t\right)+K_{I}e\left(t\right)+K_{D}\ddot{e}\left(t\right)$$

Substituting Equation (6) into Equation (8) results in the following:

(9) $$\dot{s}\left(t\right)=K_{P}\dot{e}\left(t\right)+K_{I}e\left(t\right)+K_{D}\left[{\ddot{q}}_{d}\left(t\right)-\ddot{q}\left(t\right)\right]$$

According to Equation (2),

(10) $$\ddot{q}={D_{0}}^{-1}\left(q\right)\left[\tau -C_{0}\left(q,\dot{q}\right)-G_{0}\left(q\right)+N\left(q,\dot{q},t\right)\right]$$

Substituting Equation (9),

(11) $$\dot{s}\left(t\right)=K_{P}\dot{e}\left(t\right)+K_{I}e\left(t\right)+K_{D}\left\{{\ddot{q}}_{d}\left(t\right)-{D_{0}}^{-1}\left(q\right)\left[\tau -C_{0}\left(q,\dot{q}\right)-G_{0}\left(q\right)+N\left(q,\dot{q},t\right)\right]\right\}$$

Let $\dot{s}\left(t\right)=0$; the equivalent control law can then be expressed as

(12) $$\tau_{eq}=D_{0}\left(q\right)\left\{\left[K_{P}\dot{e}\left(t\right)+K_{I}e\left(t\right)\right]/K_{D}+{\ddot{q}}_{d}\left(t\right)\right\}+C_{0}\left(q,\dot{q}\right)\dot{q}+G_{0}\left(q\right)-N\left(q,\dot{q},t\right)$$

The exponential reaching law can be written as follow:

(13) $$\dot{s}=-\varepsilon \mathrm{sgn}s-ks,\varepsilon >0,k>0$$

Reaching law acts as a bridge between the connection control algorithm and the mechanical arm, which plays an important role in resolving the conflict between chattering and control effect, this paper is based on the classical exponential approach law to improve the system on the basis of good dynamic characteristics, to reduce the response time to the sliding surface. The parameter *k* in the exponential approach law is responsible for regulating the approach rate, while the parameter ε is the main means to optimize the system vibration and ensure the system has good dynamic characteristics.

**Figure 1:**
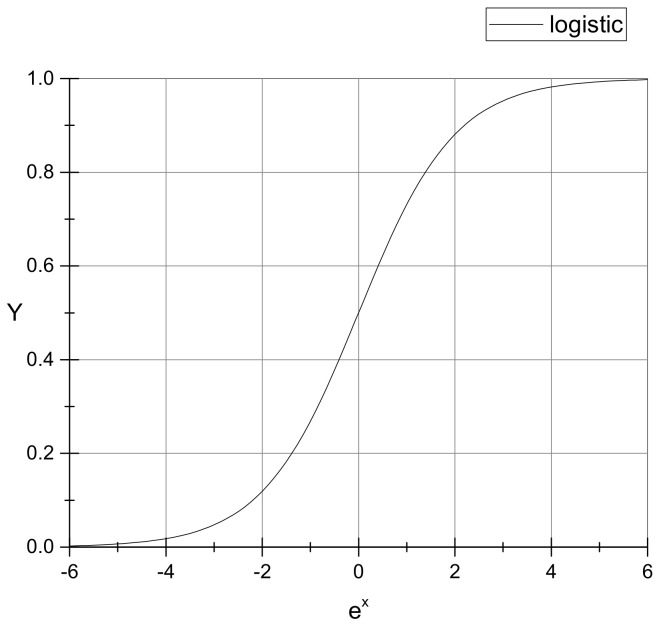
Logistic function curve: This is a standard logistic function, and it has a typical “S” shape (sigmoid curve).

The logistic function is an essential class of activation functions in neural networks, as shown in Figure 1, and it is characterized by smoothness, strict monotonicity, and saturation. A sliding mode controller based on the Logistic function was designed in this study; the exponential reaching law was adopted, and the aim is to improve a defect of the traditional sliding mode controller. This defect is the lack of smoothness at the critical value. The exponential reaching law is also characterized by a fast response and good dynamic quality.

The general form of the Logistic function is

(14) $$Logistic\left(x,A_{1},A_{2},x_{0},p\right)=A_{2}+\frac{A_{1}-A_{2}}{1+{\left(\frac{x}{x_{0}}\right)}^{p}}$$

In order to simplify the expression of the equation, it can be assumed that:

(15) $$A_{1}=1,A_{2}=0,x_{0}=1,p=-1$$

Where, as an independent parameter variable, $e^{x}$ in Equation (16):

(16) $$Logistic\left(e^{x},1,0,1,-1\right)={\left(1+e^{-x}\right)}^{-1}$$

In the control process, suppose that $t_{0}$ satisfies $e(t_{0})=0$, $\dot{e}\left(t_{0}\right)=0$, and $\int_{0}^{t}e(\tau )d\tau =0$.

According to Equation (7),

(17) $$s\left(t_{0}\right)=K_{P}e\left(t_{0}\right)+K_{I}\int_{0}^{t_{0}}e\left(\tau \right)d\tau +K_{D}\dot{e}\left(t_{0}\right)=0$$

It is proved that the algorithm can satisfy the necessary and sufficient conditions of existence and accessibility, the switching control law is

(18) $$\tau_{s}=\varepsilon \mathrm{sgn}s+ks,\varepsilon >0,k>0$$

**Figure 2:**
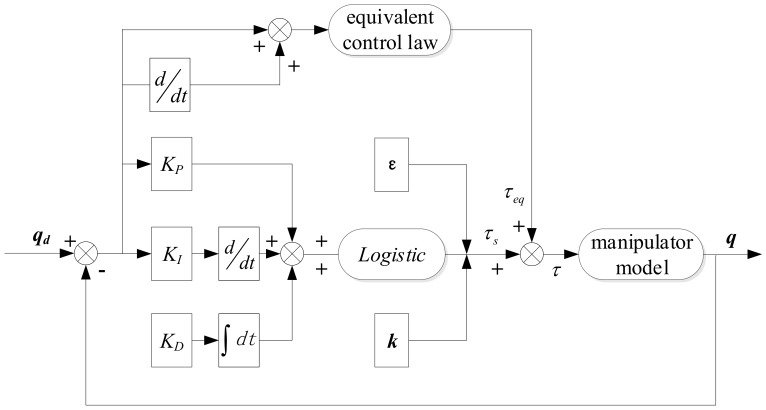
Block diagram of the adaptive gravity compensation architecture of a space robotic manipulator.

The switching control law here can compensate for the uncertainty in the system and ensure the robustness of the system. The system control block diagram is shown in Figure 2.

Substituting Equation (15) into (18), the following is obtained:

(19) $$\tau_{s}=\varepsilon {\left(1+e^{-s}\right)}^{-1}+ks,\varepsilon >0,k>0$$

The total control torque of the system is the sum of Equation (12) and Equation (19):

(20) $$\tau =\tau_{eq}+\tau_{s}$$

The stability proof of the gravity pretreatment part is given in the following stability analysis.

#### 2.2.2. Friction Compensation

The primary purpose of the spatial disturbance observer (SDO) is to (1) reduce the extra unknown disturbance torque by as much as possible while not adding any sensors and (2) estimate the friction in the system on the basis of observation results to enable friction compensation. Although disturbance observer technology is widely used in the control of manipulators, at the present stage, most disturbance observers are based on linear models or linear systems. The spatial robotic manipulator presented in this paper is a highly coupled nonlinear system, so existing disturbance observers cannot be used for control.

According to the description in the previous section, the disturbance torque can be represented by

(21) $$d=N\left(q,\dot{q},t\right)=D\left(q\right)\ddot{q}+C\left(q,\dot{q}\right)\dot{q}+G\left(q\right)-\tau$$

The estimation of the disturbance moment can be expressed in the following form. The objective of the process described in this section is to design a spatial disturbance observer to estimate the disturbance moment.

(22) $$\dot{\hat{d}}=-L\left(q,\dot{q}\right)\hat{d}+L\left(q,\dot{q}\right)\left(D\left(q\right)\ddot{q}+C\left(q,\dot{q}\right)\dot{q}+G\left(q\right)-\tau \right)$$

$\dot{\hat{d}}$ Contrary to other observers, the presented observer of the disturbance torque represents the friction force in friction compensation. In another case, single joint control, it represents the unmodeled dynamics. A direct result of the item in the previous section is gravity pretreatment, so the design of the space disturbance observer in this chapter focuses on friction as the primary source for modeling and analysis.

First, the observer error is defined:

(23) $$e_{2}\left(t\right)=d-\hat{d}$$

Combining Equation (23) with the observer Equation (22) yields

(24) $${\dot{e}}_{2}\left(t\right)=\dot{d}-\dot{\hat{d}}=L\left(q,\dot{q}\right)\hat{d}-L\left(q,\dot{q}\right)d$$

The auxiliary variables are defined as

(25) $$\dot{z}=\hat{d}-p\left(q,\dot{q}\right)$$

where $z\in R^{2}$, and the designed function vector $p(q,\dot{q})$ is to be determined.

(26) z˙=^˙d−dp(q,q˙)dt=^˙d−∂p(q,q˙)∂q∂p(q,q˙)∂qq˙q¨

Let the function $p(q,\dot{q})$ in Equation (22) be given by the following nonlinear equation:

(27) $$L\left(q,\dot{q}\right)D\left(q\right)\ddot{q}=\left[\begin{array}{cc} \frac{\partial p(q,\dot{q})}{\partial q} & \frac{\partial p(q,\dot{q})}{\partial q} \end{array}\right]\left[\frac{\dot{q}}{\ddot{q}}\right]$$

Therefore, the auxiliary variable z designed in this study can be expressed as follows:

(28) $$\dot{z}=-L\left(q,\dot{q}\right)z+L\left(q,\dot{q}\right)\left(C\left(q,\dot{q}\right)\dot{q}+G\left(q\right)-p\left(q,\dot{q}\right)-\tau \right)$$

Hence, the SDO is given by:

(29) $$\hat{d}=-L\left(q,\dot{q}\right)z+L\left(q,\dot{q}\right)\left(C\left(q,\dot{q}\right)\dot{q}+G\left(q\right)-p\left(q,\dot{q}\right)-\tau \right)+L\left(q,\dot{q}\right)$$

The Novel Friction Model is based on the LuGre model. In the traditional LuGre model,

(30) $$\dot{z}=v-\frac{\left|v\right|}{g(v)}z$$

(31) $$f=\sigma_{0}z+\sigma_{1}\dot{z}+\sigma_{2}v$$

where *z* represents the state variable observed by the spatial disturbance observer, *v* represents the velocity term, g(v) represents the function related to velocity, which is described in detail in [3], $\sigma_{0}$ represents the equivalent stiffness of the dynamic relationship between force and displacement when the velocity is reversed, $\sigma_{1}$ represents the viscous friction coefficient that is observed slightly, and $\sigma_{2}$ represents the viscous friction coefficient. Although the traditional LuGre model can adequately describe the friction phenomenon in most cases, it does not explain the pre-sliding friction when the velocity is reversed, especially in the space microgravity environment.

The LuGre model can be rewritten as follows:

(32) $$f=F_{h}\left(z\right)+\sigma_{1}\dot{z}+\sigma_{2}v$$

$F_{h}\left(z\right)$ is the hysteresis friction force [51], which is the part of the friction force exhibiting hysteretic behavior. It is static hysteresis nonlinearity with nonlocal memory; this property is in contrast to the action of hysteresis nonlinearities with local memory, where the past exerts its influence on the future through the current value of the output only [52].

(33) $$F_{h}\left(z\right)=F_{b}+F_{d}\left(z\right)$$

where $F_{b}$ is the beginning of a transition curve, and $F_{d}\left(z\right)$ is the current transition curve at a particular time. From the current hysteresis transition curve and the current velocity, the nonlinear state equation can be obtained as shown in [53].

(34) $$\dot{z}=v\left(1-sign\left(\frac{F_{d}\left(z\right)}{S\left(v\right)-F_{b}}\right)*{\left|\frac{F_{d}\left(z\right)}{S\left(v\right)-F_{b}}\right|}^{n}\right)$$

For constant velocity, $\dot{z}=0$,

(35) $$S\left(v\right)=F_{d}\left(z\right)+F_{b}$$

(36) $$f=S\left(v\right)+\sigma_{2}v$$

S(v) determines the low-speed characteristic of the system, and $\sigma_{2}v$ plays a significant role in the higher speed of the system. According to [54], it is known that

(37) $$S\left(v\right)=F_{c}+\left(F_{s}-F_{c}\right)*c^{-{\left(v/v_{s}\right)}^{\delta }}$$

where $F_{c}$ is the Coulomb friction, $F_{z}$ is the static friction, $v_{s}$ is the Stribeck velocity, and δ is an arbitrary exponent.

The main idea in this part is to pretreat the gravity term and compensate for the friction in the dynamic model of the manipulator. To achieve more accurate trajectory tracking, a hierarchical closed-loop control framework, including a primary system and two auxiliary closed-loop systems, was adopted. Cartesian position control is used as the main loop, and gravity pretreatment and friction compensation are used as the secondary loop policy. The sliding mode control strategy based on the sigmoid function is adapted for the gravity term, and the friction force is regarded as the disturbance. Then, the disturbance is observed by the spatial disturbance observer (SDO).

**Figure 3:**
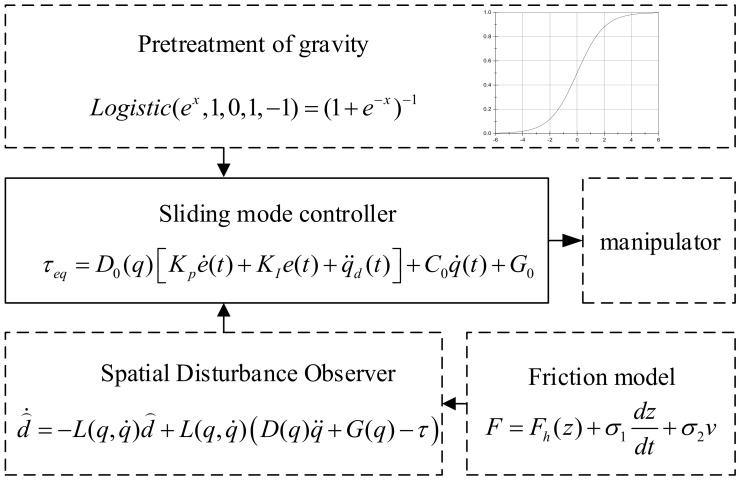
Whole block diagram.

Considering the disturbance observer, gravity term pretreatment, sliding mode controller and friction compensation, the complete control flow of the space manipulator is shown in the Figure 3.

This control framework can minimize the influence of the microgravity environment and meet the stability requirements.

### 2.3. Stability Analysis

#### Stability

Inspired by literature [55] and literature [56], the system stability is proved as follows

**Theorem** **1.** *For the manipulator system (2) and (3), the controller is given by Equation (20). Then, the manipulator system is asymptotically stable.*

**Proof.**  Let the Lyapunov function be defined by Equation (38):

(38) $$V=\frac{1}{2}s^{T}Ds$$

(39) $$\dot{V}=\frac{1}{2}s^{T}\dot{D}s+s^{T}D\dot{s}$$

For any vector ξ,

(40) $$\xi^{T}\left(\dot{D}-2C\right)\xi =0$$

where *D* denotes the inertia matrix and *C* denotes the centrifugal force and the Coriolis force of the Equation (1), Therefore

(41) $$\dot{V}=s^{T}\left(D\dot{s}+Cs\right)$$

Substituting Equation (9) into (41),

(42) $$\dot{V}=s^{T}\left\{D\left[K_{P}\dot{e}+K_{I}e+K_{D}\left({\ddot{q}}_{d}-\ddot{q}\right)\right]+Cs\right\}$$

Substituting Equations (7) and (20) into (42),

(43) $$\dot{V}=s^{T}\left\{D\left(K_{P}\dot{e}+K_{I}e+K_{D}{\ddot{q}}_{d}\right)-K_{D}\left(\tau_{eq}+\tau_{s}-C_{0}-G_{0}+N\right)+C\left[K_{P}e+K_{I}\int_{0}^{t}e\left(\tau \right)d\tau +K_{D}\dot{e}\right]\right\}$$

Simplifying Equation (12),

(44) $$\dot{V}=-s^{T}\tau_{s}$$

Substituting Equation (19) into (44),

(45) $$\dot{V}=-s^{T}\varepsilon {\left(1+e^{-s}\right)}^{-1}-s^{T}ks\leq 0$$

Q.E.D □

## 3. Experiment and Results

### 3.1. Experimental Platform

#### 3.1.1. Overview of Space Robotic Arm-Hand System

**Figure 4:**
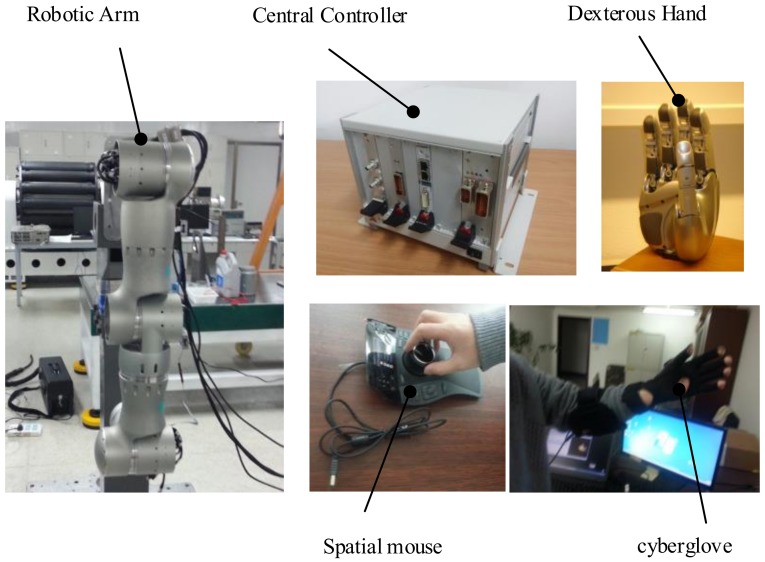
Prototype of space manipulator system.

Space manipulator system is responsible for on-orbit servicing missions, such as space capture tasks and maintenance of electrial connection, so the core of system is a 6-DOF humanoid robotic arm. At the end of the robotic arm connection is a humanoid multi-finger dexterous hand with quick-change flange. The dexterous hand wrist has a camera that captures images of objects in the cabin in real time. These entities are controlled by a central controller. In order to achieve the operation on the redundancy, the system also configures a teleoperation system which can be operated by astronauts in real-time. The robotic arm and dexterous hands integrate a large number of sensors, which make the whole system with system highly intelligent. Composition of robotic arm-hand system is shown in Figure 4.

#### 3.1.2. Robotic Arm

Robotic arm is composed of modular joints, a total of six degrees of freedom, in order to simplify structure design. The elbow and wrist joints are the same. Motors, sensors and electrical configurations of each joint are identical. The benefit is that, once a joint is damaged, the replacement can be done quickly, joint integration with the position, torque, temperature, electric current and other sensors, as shown in Table 1.

These sensors for robotic arm control provide a hardware foundation, will also enhance the level of intelligence.

#### 3.1.3. Dexterous Hand

Multi-finger dexterous hand with HIT/DLR II five fingers dexterous hand, the appearance and function of the structure are more humanization, integrated torque sensor, position sensor and temperature sensor, and integration within the fingers, smart grasping ability, make it is suitable for all kinds of tasks of on-orbit capture and maintenance.

#### 3.1.4. Teleoperation System

The robotic arm system is also equipped with a remote operating system as a backup operating system for the autonomous control of the arm. The astronauts can operate the arm to complete the task through two input devices, namely Spatial mouse and the cyberglove.

#### 3.1.5. Central Controller

The central controller is mainly used to complete the planning and control of manipulator and dexterous hand in tasks, and it is also responsible for power management, network communication and other related functions.

#### 3.1.6. Visual System

**Figure 5:**
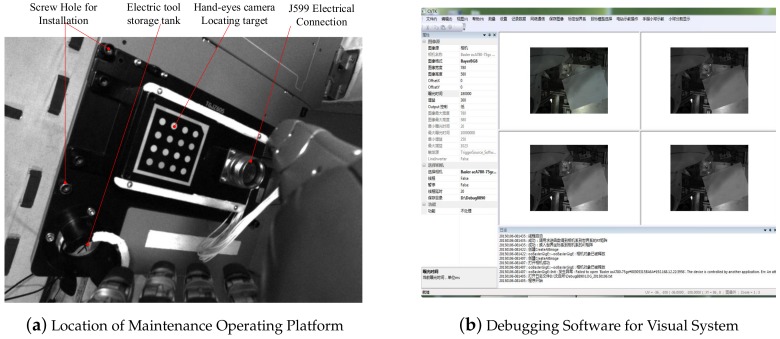
(**a**) illustrates how to locate the maintenance operation platform by using the target of the hand-eye camera. There are J599 electrical connectors and hand-held electrical cameras on the maintenance operation platform. The tool storage tank (also a maintenance task limited to space; the following focuses on the maintenance operation of the J599 electrical connector) and installation of screw holes are shown. (**b**) is the hand-eye camera debugging software, which adjusts the white balance.

The visual system is mainly equipped with three cameras, two of which are suspended from the ceiling of the laboratory module, one of which is mounted on the connecting flange of the manipulator and the dexterous hand. The collected images are used as the input to the capture task and processed by the central controller. Location of Maintenance Operating Platform and Debugging software is show in Figure 5.

**Figure 6:**
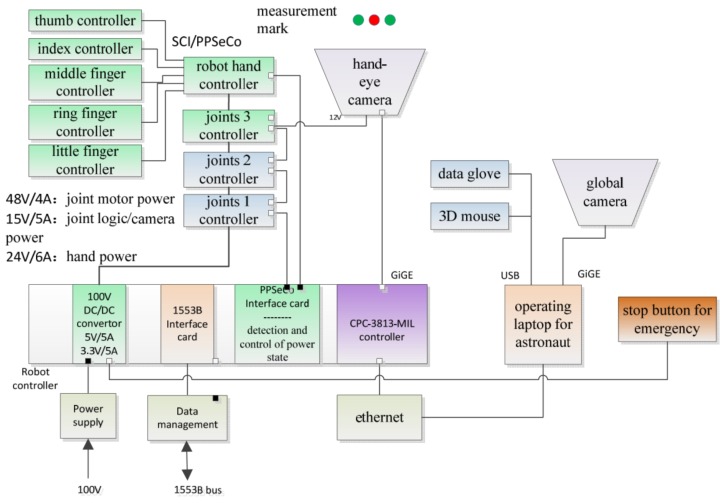
System control block diagram.

Generally speaking, the experimental platform of the humanoid manipulator system consists of three functional components: (1) the body of the manipulator system comprising a 6-DOF humanoid manipulator and a multi-fingered dexterous hand; (2) the control system consisting of a central controller, a teleoperation controller, and its auxiliary controller; and (3) the visual measurement system consisting of a global camera and a hand-eye camera. The control architecture of the experimental platform of the humanoid manipulator system is shown in Figure 6. The external interface of the central controller receives a 100 V upstream power supply from a TG2 experimental cabin. The system is connected to a TG2 digital tube system through the 1553B bus, receives ground instructions, and returns telemetry data. The central controller receives backup control instructions from the teleoperation system from above and below the main body of the manipulator system to control the manipulator and the dexterous hand’s performance of tasks. The teleoperation system consists of a teleoperation controller and an auxiliary controller, which includes a data glove and a three-dimensional mouse, and is used to plan the upper trajectory of the task.

### 3.2. Design of Experimental System

In this section, two typical on-orbit experiments are presented to verify the effectiveness and superiority of the proposed controller. First, the designed capture experiment is presented; in this task, a ball floating in space is captured by the manipulator system. Then, the designed on-orbit maintenance experiment is presented; in this experiment, the J599 electrical connector is disassembled by the manipulator system.

#### 3.2.1. Capture Task

**Figure 7:**
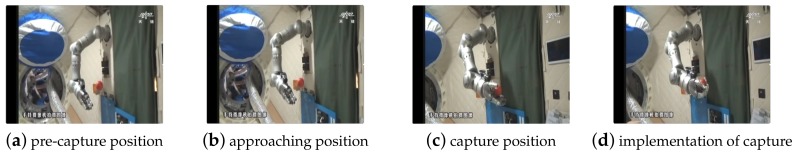
(**a**) shows the ball in the pre-capture position. At this time, the manipulator starts to move by the global camera servo. (**b**) shows that the manipulator is already in the approximate grasp position. At this time, it enters the field of view of the hand-eye camera. The position information of the ball is provided by the hand-eye camera for servo control. (**c**) shows that the manipulator has reached the graspable position. The hand-eye camera and the global camera are simultaneously providing graspable confidence. The degree parameter in (**d**) shows that the manipulator has received the capture command from the dual camera. It performs the capture operation and accomplishes the task.

The capture task is shown in Figure 7a–d. The astronaut releases the ball in free space, and the ball floats freely. The manipulator system uses the visual system to make the transition from the pre-capturing position to the capturing position.

**Figure 8:**
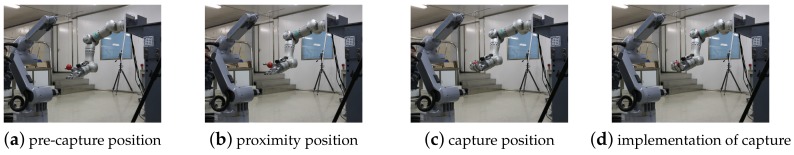
In the ground simulation experiment of the capture mission, each link is the same as that in the space experiment, in which the free speed of the pellet is strictly limited.

To simulate the wind speed resulting from the ventilation system in the experimental cabin, the offline programming of industrial robots is used to guide the ball, and the four stages of the acquisition task in different wind speeds are verified which is shown in Figure 8. It is worth noting that the load of the manipulator is equal to the sum of the dexterous hand and the mass of the ball at the moment when the ball is captured by the manipulator system, which leads to a change in the dynamic model of the manipulator.

#### 3.2.2. Maintenance Task

**Figure 9:**
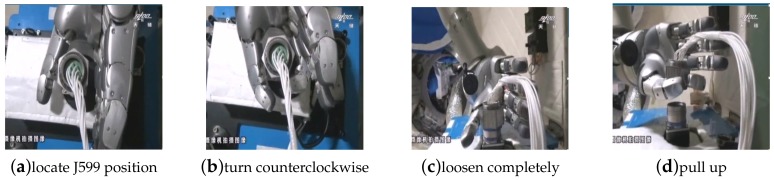
(**a**) shows the location of the electrical connector by locating the target of the maintenance operation platform. (**b**) shows that the dexterous hand envelops the J599 electrical connector and rotates counter-clockwise. (**c**) shows that the electrical connector is wholly separated from the connector parent. (**d**) shows that the electrical connector is pulled up to complete the maintenance task.

The maintenance task in this study refers to the disassembly of the J599 electrical connector from the installation position by using the manipulator arm system, and it is different from the capture task. This task is a contact task which is shown in Figure 9. It requires the precise force control of the manipulator arm given the circumstance that the rotating axis of the manipulator does not completely match the geometric axis of the electrical connector. This is to ensure that the electrical connector is completely secured with screws and, at the same time, cannot be used to control the mechanical arm, as smart hands lead to damage.

**Figure 10:**
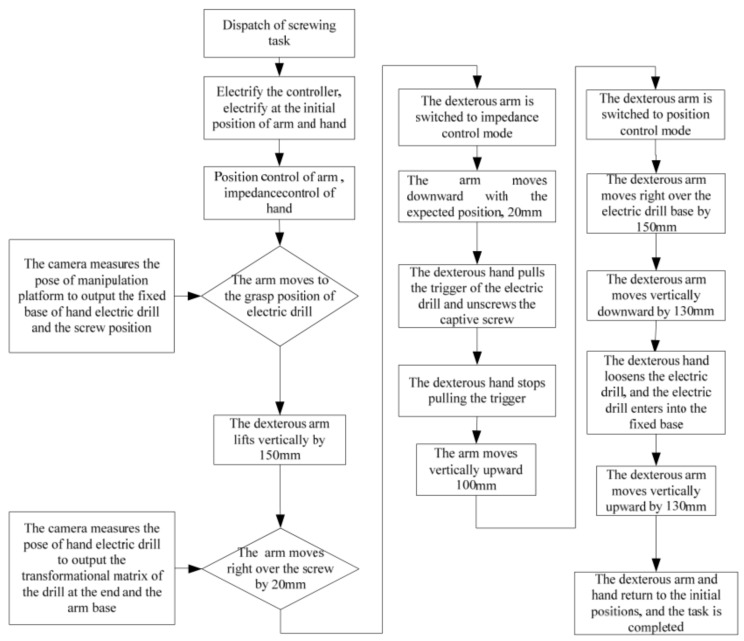
Operation Flow of Rotating the J599 Electric Connector by the Manipulator Hand System.

The operation process as shown in Figure 10. This task mainly uses robotic arm hand system, rotary screw electric connector J599, and separates the electric connector from the maintenance operation platform. First, the hand-eye camera obtains the position of the maintenance operation platform and electric connector. Under the condition of orbit. Under microgravity conditions, the maintenance operation platform will deform, so the position of the electrical connector cannot be obtained in advance. Instead, the position of the electrical connector in the camera frame is transferred to the tool frame of the manipulator. When we get the position information of the electric connector, the dexterous hand will move to the electric connector directly, and then falls down slowly close to the electric connector. The thumb and index finger grip the electric connector, monitor fingers torque information, ensure the dexterous hand can hold the connector without damaging the fingers. Screw it 4 times, 120${}^{{}^{\circ}}$ at a time, and then the connector can be pulled out.

Due to robotic arm configuration’s constraints, it doesn’t allow the dexterous hand to rotate the electric connector continuously. Therefore, the decomposition action is adopted. The sequence is, rotary, loosen, back to origin place, spiral twist 60${}^{{}^{\circ}}$ at a time, a total of the rotary screw four times. The dexterous hand and electric connector are the rigid body, a point of contact between the finger and the connector causes the dexterous hand to rotate once and the connector to rotate less than once, so four laps are needed, it can make the electric connector completely away from the constraints of the socket.

**Figure 11:**
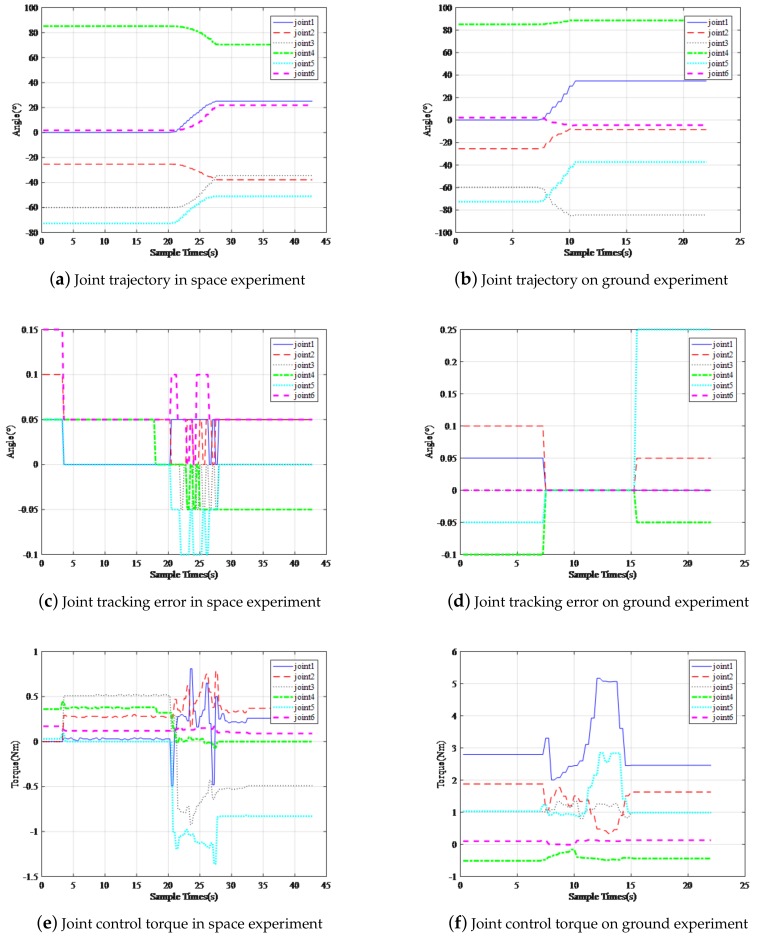
(**a**,**b**) represent the trajectories of the end of the manipulator in space and on the ground, respectively. As described above, the ground simulation experiment is guided by offline programming of the industrial robot. Therefore, the trajectories are not completely consistent with those in the space experiment, but the experimental results are not affected. (**c**,**d**) show the joint angle errors in the acquisition task. It can be seen that the errors are controlled in a small range. Within this range, (**e**,**f**) show the joint moment in the capture task. In the ground experiment, the joint moment is markedly larger than that in the space experiment.

It describes the state of the robotic arm on-orbit and on ground in Figure 11. The controller proposed in this paper is applied, it should be pointed that, these track records the movement of the manipulator from the fixed position (zero-position configuration) to the pre-operation position, in Figure 11a,b of the two pictures. The target location of the ground electric connector is not quite the same with that on-orbit. Therefore, the angle of each joint is slightly different, but it does not affect the interpretation of the results in this paper. It can be seen from Figure 11c,d that, the tracking error on-orbit is between −0.1${}^{{}^{\circ}}$ and 0.15${}^{{}^{\circ}}$, and the tracking error on ground is between −0.1${}^{{}^{\circ}}$ and 0.25${}^{{}^{\circ}}$, good repeatable positioning accuracy is maintained, and It can be seen from Figure 11e,f that, the joint torque maintains within 1.5 N on-orbit state, while on ground state, all the joint torque maintain within 5 N, it meets the mission requirements of the manipulator, but there is still room for improvement.

**Figure 12:**
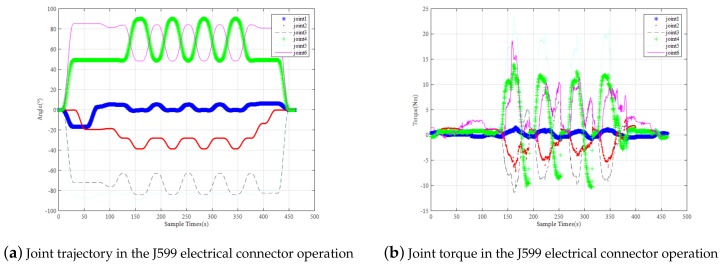
Joint Trajectory and Joint Torque of Robot Arm in the Experiment of Turning the J599 Electric Connector.

**Figure 13:**
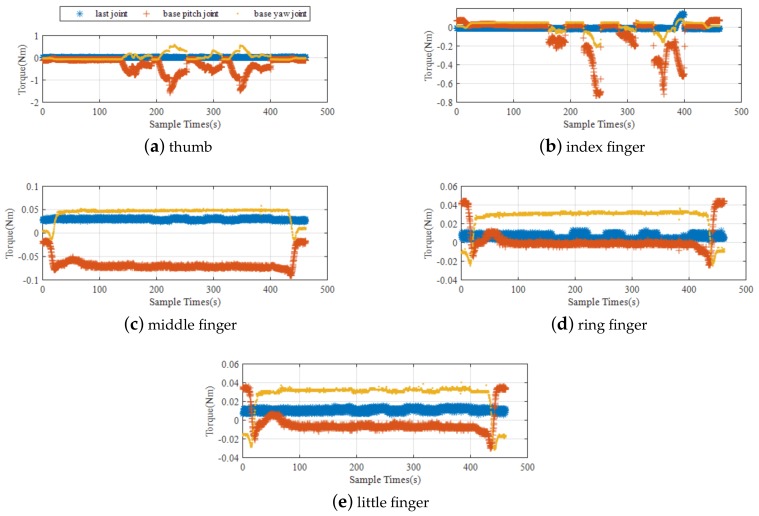
Torque of each finger joint.

In the process of twisting the J599 electrical connector, the joint angle and joint torque of the manipulator are shown in Figure 12, both are within the range of the robotic arm. The joint moment of the dexterous hand is shown in Figure 13. It can be seen that during the whole task, only the thumb and index finger bear the twisting torque, the maximum moment for the thumb is 1.5 N and the maximum moment for the index finger is 0.7 N. Also within the range of a dexterous hand.

## 4. Conclusions

We proposed an adaptive controller that can be used in a microgravity environment. The controller design is based on a space disturbance observer from the viewpoint of disturbance suppression. Sliding mode control is used to preprocess the gravity term. An adaptive compensation scheme based on the LuGre model is proposed to compensate for the friction. Capture experiments and maintenance experiments were designed and implemented to verify the controller. The results show that the proposed controller can guarantee the control accuracy with an error range of 0.15 degrees in the capture experiment, and the contact torque of the dexterous finger can be controlled with an error range of 1 Nm in the maintenance experiment. This level of control protects the dexterous hand from damage while ensuring the completion of the task.

## Figures and Tables

**Table 1:** Sensor configuration of the arm joint.

No.	Sensor Type	Amount of Joints	Measurement Principle
1	Joint torque	1	Strain
2	Joint position	1	Magnetic encoder
3	Motor end position	1	Rotary transformer
4	Motor position	3	Digital hall
5	Current sensor	2	Resistance drop
6	Temperature sensor	1	Thermometer

## Abbreviations

The following abbreviations are used in this manuscript:

PPSeCo	Point-to-Point High Speed Serial Communication
FPGA	Very-High-Speed Integrated Circuit Hardware Description Language
SDO	Space Disturbance Observer
DSP	Digital Signal Processor
SMC	Sliding-mode Control
